# A Systemic Investigation of Genetic Architecture and Gene Resources Controlling Kernel Size-Related Traits in Maize

**DOI:** 10.3390/ijms24021025

**Published:** 2023-01-05

**Authors:** Cheng Wang, Huangai Li, Yan Long, Zhenying Dong, Jianhui Wang, Chang Liu, Xun Wei, Xiangyuan Wan

**Affiliations:** 1 Research Center of Biology and Agriculture, Shunde Innovation School, School of Chemistry and Biological Engineering, University of Science and Technology Beijing, Beijing 100083, China; 2 Beijing Engineering Laboratory of Main Crop Bio-Tech Breeding, Beijing International Science and Technology Cooperation Base of Bio-Tech Breeding, Zhongzhi International Institute of Agricultural Biosciences, Beijing 100192, China

**Keywords:** grain yield, quantitative trait locus (QTL), quantitative trait nucleotide (QTN), genome-wide association study (GWAS), gene, molecular breeding, maize

## Abstract

Grain yield is the most critical and complex quantitative trait in maize. Kernel length (KL), kernel width (KW), kernel thickness (KT) and hundred-kernel weight (HKW) associated with kernel size are essential components of yield-related traits in maize. With the extensive use of quantitative trait locus (QTL) mapping and genome-wide association study (GWAS) analyses, thousands of QTLs and quantitative trait nucleotides (QTNs) have been discovered for controlling these traits. However, only some of them have been cloned and successfully utilized in breeding programs. In this study, we exhaustively collected reported genes, QTLs and QTNs associated with the four traits, performed cluster identification of QTLs and QTNs, then combined QTL and QTN clusters to detect consensus hotspot regions. In total, 31 hotspots were identified for kernel size-related traits. Their candidate genes were predicted to be related to well-known pathways regulating the kernel developmental process. The identified hotspots can be further explored for fine mapping and candidate gene validation. Finally, we provided a strategy for high yield and quality maize. This study will not only facilitate causal genes cloning, but also guide the breeding practice for maize.

## 1. Introduction

**Figure 1:**
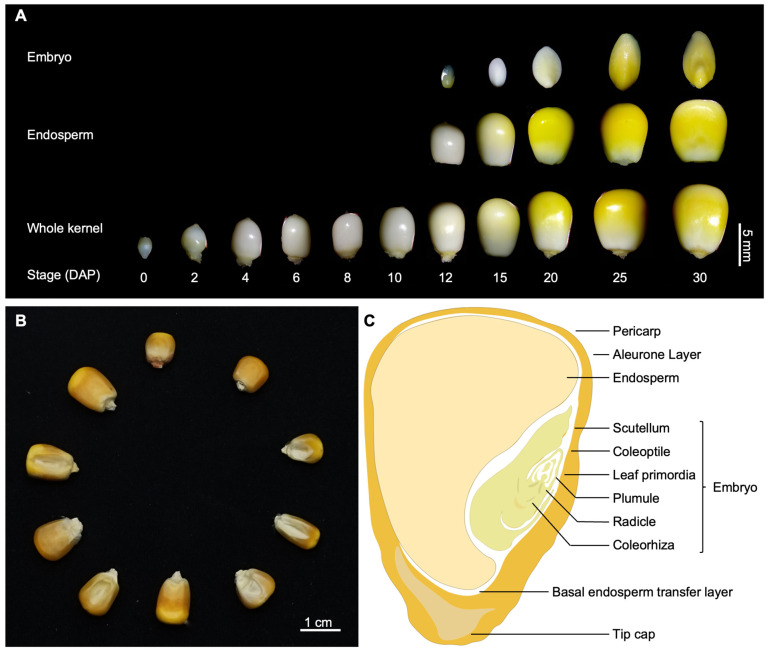
Illustration of maize kernel developmental period and structure. (**A**) Overview of kernel developmental stages in maize inbred line B73 (DAP, days after pollination). (**B**) Seed size diversity in different maize lines. (**C**) Diagram of maize kernel structure.

The maize kernel, as in other crops, initially develops from the double fertilization event [1], which leads to the formation of the diploid embryo and triploid endosperm, and finally, grows into a mature grain (Figure 1A,B). The developing maize kernel consists of three major distinct compartments: embryo, endosperm, and pericarp (Figure 1C), wherein the embryo and endosperm are wrapped by the pericarp. The embryo, representing the generation of a new plant, is the most critical component of the seed. Plant embryogenesis undergoes a sequential of partitioning events to produce a fully developed embryo with scutellum (cotyledon), coleoptile, leaf primordia, plumule, radicle, and coleorhiza (Figure 1C) [2,3,4]. Endosperm development starts with the fertilization of the central cell [5]. Following endosperm cellularization, the central cell differentiates into four cell types: aleurone layer (AL), basal endosperm transfer layer (BETL), starchy endosperm (SE), and embryo-surrounding region (ESR) (Figure 1C). At the later differentiation stage, the four main cell types further differentiate and form new cell types: sub-aleurone, conducting zone (CZ), and basal intermediate zone (BIZ) [6,7]. Each cell type has distinct characteristics in cellular morphology, gene expression pattern, and biological function [7,8]. Mostly, defects in either embryo or endosperm development would affect kernel size and eventually, lead to yield loss.

As the most relevant yield factor, maize kernel is the main target for breeding. Kernel morphology is crucial in determining kernel size and yield. Maize kernel has variable types of phenotypic variation (Figure 1B). For example, *defective kernel* mutants (*Dek*) are caused by abnormal embryo development and impairments of starch and protein synthesis in the endosperm [9,10]. Compared to wild type, *small kernel* mutants (*Smk*) have smaller kernels and delayed kernel development [11]. In *embryo specific* (*Emb*) mutants, endosperm develops normally and the embryo shows more or less severe aberrations [12], which is opposite to that in *endosperm specific* mutants (End) [13]. The *empty pericarp* (*Emp*) mutants exhibit empty pericarp or papery seeds in mature ears [14], *opaque/floury* mutants refer to those with a reduction in the content of zein in the endosperm [15], and *shrunken* mutants refer to those with the starch-deficient phenotype [16]. The remarkable diversity of kernel morphology in maize provides excellent research systems to explore the underlying genetic basis and molecular mechanisms of kernel development.

Grain yield is one of the most significant and complex quantitative traits in maize. It has been demonstrated to be affected by multiple factors, including genetic, environmental, and nutritional factors, and also their interaction with each other [17,18]. Grain size-related traits are crucial determinants for crop yield in cereals, including rice [19,20,21,22,23], wheat [24,25,26], and maize [27,28,29]. Four major kernel size-related traits, kernel length (KL), kernel width (KW), kernel thickness (KT), and hundred-kernel weight (HKW), are the most important characteristics that determine grain yield in maize. Besides, these traits are also found to be significantly related to the nutrient contents of maize seeds [30] and employed as the essential criteria for evaluating early seeding vigor [31]. Genetic dissection of kernel size-related traits will accelerate the understanding of kernel development, which in turn will facilitate efficient improvement of maize yield.

Quantitative trait locus (QTL) analysis, known as QTL mapping, is a statistical method that links phenotypic variation to genetic maps. Genome-wide association assay (GWAS) is another powerful approach for identifying genomic regions and genetic variants associated with phenotypes. Over recent decades, hundreds of QTLs and thousands of quantitative trait nucleotides (QTNs) have been identified for kernel size-related traits in maize [6,32,33]. Compared to massive QTLs and QTNs reported, only a relatively small portion was fine mapped and few genes were further identified [33]. Additionally, the utilities of these QTLs and QTNs are limited by many factors, including different genetic backgrounds evaluated in diverse environments following distinct methods for detection [32,34]. Thus, an integration analysis of QTLs and QTNs based on different results can help to identify stable QTLs and QTNs with significant effect.

Recent studies have identified a number of genes as key kernel size regulators, which are involved in multiple signaling pathways, for instance, post-transcriptional regulation of mitochondrial and chloroplast genes, starch synthesis, secondary metabolic pathways, cell cycle regulation, sugar/amino acid transport, the phytohormone signaling pathway, and transcriptional regulation [6,33]. However, our understanding of the mechanisms of kernel development is still poor and full of gaps, so more efforts are required to identify new genes controlling kernel size and advance our knowledge of kernel development.

In this study, we first carried out a bibliometric analysis of maize yield research over the past decades, which provided an overview of the publications in this field based on the quantitative and performance assessment and predicted future research trends through the hotspot analysis. Then, expression pattern and gene ontology (GO) enrichment analyses were conducted for reported genes regulating kernel size in maize. After that, the published QTLs and QTNs data were collected to identify clusters associated with kernel size-related traits on the whole genome, which were further integrated to unravel the consensus hotspot regions and screen promising candidate genes for maize yield. Finally, we proposed a strategy model for producing high yield and quantity maize.

## 2. Results

### 2.1. Bibliometric Analysis of Kernel Size-Related Traits in Maize

**Figure 2:**
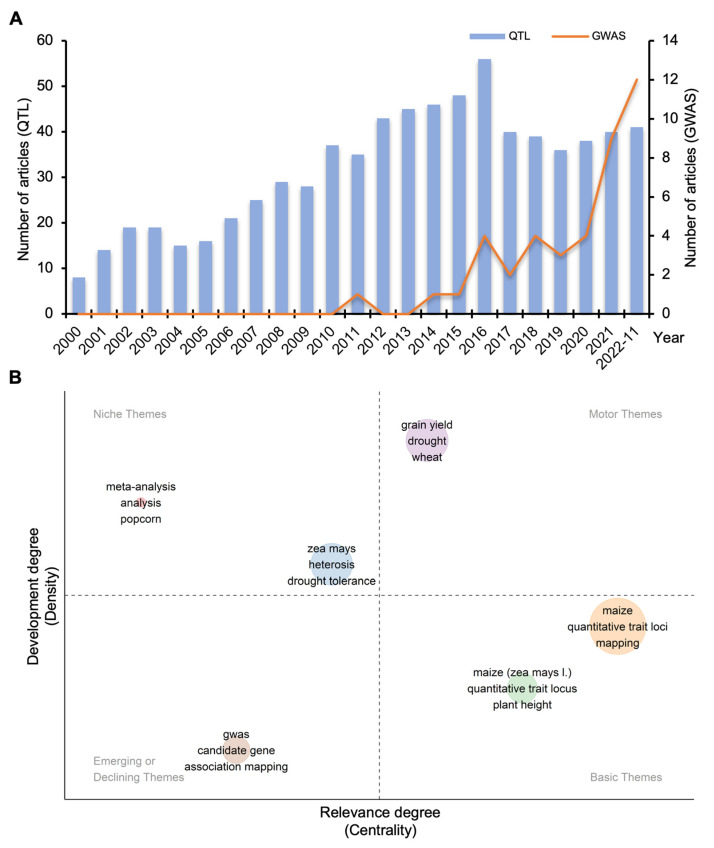
Current situation and future trend of the research of maize kernels. (**A**) The number of GWAS and QTL mapping analysis publications on kernel size from 2000 to November 2022. (**B**) Predicted hot research directions based on known research results.

As kernel size-related traits are the most directly correlative traits for grain yield in maize, they have always been popular research topics in history, especially in the past two decades. As shown in Figure 2A, the total publications in this field gradually increased since 2000, representing the high level of academic interest and popularity. It was also found that the publications on QTL mapping reached a peak in 2016 and began to grow slowly due to the development of GWAS technology. Hot research direction analysis showed that “maize”, “quantitative trait loci”, and “mapping” were the most relevant topics, and “grain yield”, “meta-analysis”, and “heterosis” might develop best in the future (Figure 2B).

**Figure 3:**
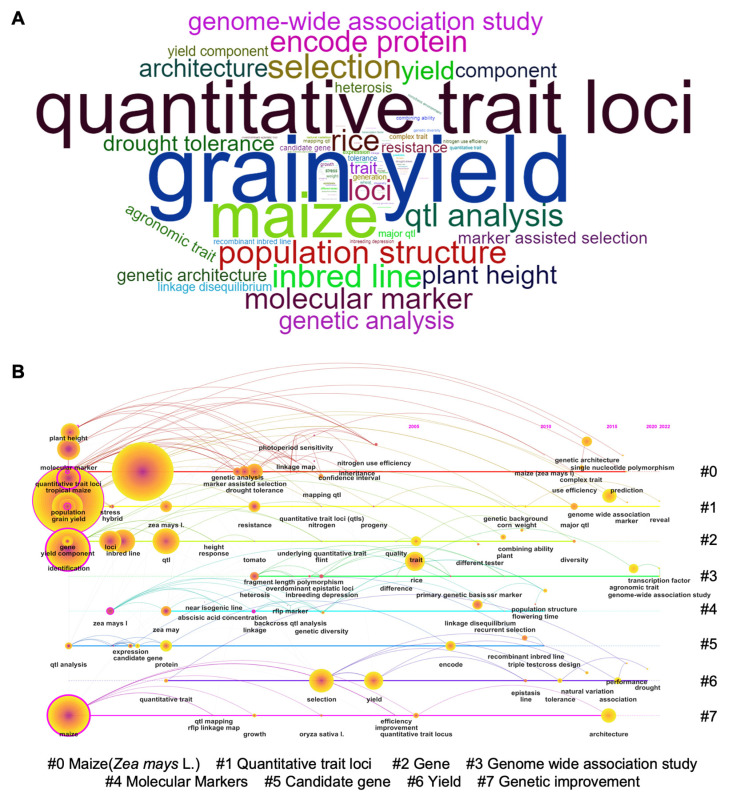
Hot topics and the knowledge structure of kernel size and seed weight in maize. (**A**) Word cloud of kernel research in maize. (**B**) Sequence diagram of eight clusters of keywords in maize kernel size-related research. Nodes are labeled with corresponding topics.

It is believed that big progress has been achieved in the research on QTL of grain yield in maize, which is consistent with the key words of high frequency and centrality analysis by bibliometric analysis (Figure 3A). Related studies mainly fall into eight clusters, and genetic analysis of yield-related traits is the focus of continuous attention. It is clear that a nonstress environment and heterosis in maize have been the research mainstreams for a long time. After systematic evolvement, the research hotspots now focus on GWAS analysis and the genetic architecture of the agronomic trait (Figure 3B).

Taken together, genetic architecture and molecular improvement of kernel size-related traits in maize remain active research fields and will be fascinating for researchers in the future.

### 2.2. Characterization of Cloned Genes Controlling Maize Kernel Size-Related Traits

To date, 132 genes have been reported to be involved in kernel development (Table 1), and a large portion of them belong to a pentatricopeptide repeat (PPR) protein family.

**Figure 4:**
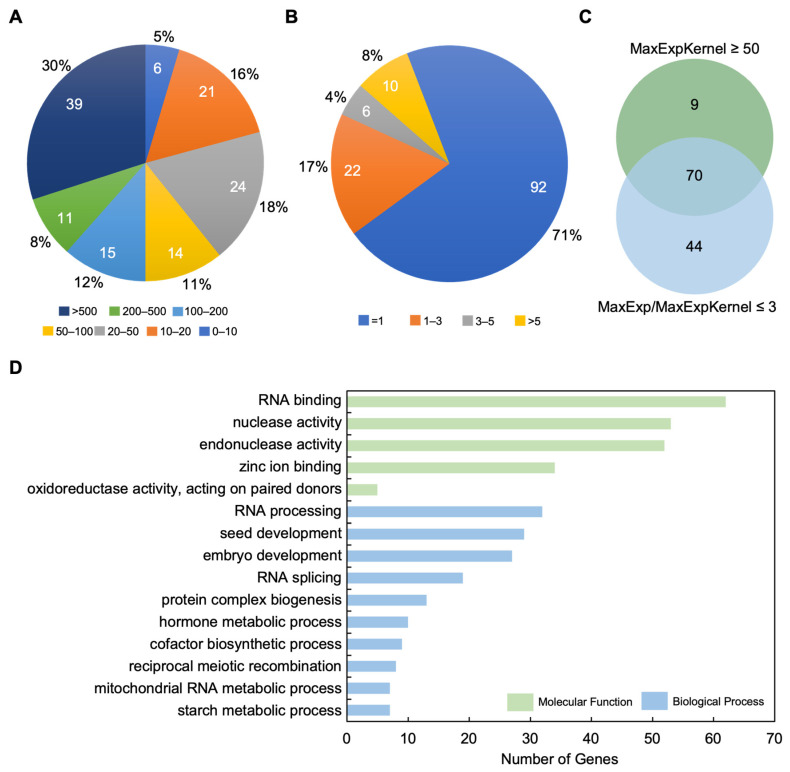
Expression pattern and GO enrichment analyses of the reported genes related to kernel size in maize. (**A**) The distribution of maximal gene expression in kernels (MaxExpKernel) among reported genes controlling kernel size-related traits. The pie chart shows the categorization of cloned genes based on the MaxExpKernel FPKM values. Sectors in different colors represent the number of genes with a distinct range of MaxExpKernel FPKM values. (**B**) The distribution of the ratios of maximal expression in all tissues (MaxExp) to MaxExpKernel. The pie chart shows the categorization of cloned genes based on the ratios of MaxExp to MaxExpKernel. Sectors in different colors represent the number of genes with a distinct range of MaxExp/MaxExpKernel values. (**C**) Venn diagram of number of genes with two expression features MaxExpKernel ≥ 50 and MaxExp/MaxExpKerenel ≤ 3. Upper green chart represents the number of cloned genes with MaxExpKernel ≥ 50, and lower blue chart represents the number of cloned genes with MaxExp/MaxExpKernel ≤ 3. (**D**) GO enrichment analysis of reported genes.

We first performed expression pattern and GO enrichment analysis with these cloned genes. Gene expression pattern analysis was carried out based on reported RNA sequencing (RNA-seq) data [156]. We collected expression data of 130 genes and no expression data were available for two genes. It was found that all of the 130 genes were expressed in kernels, suggesting a role in kernel development. Next, fragments of kilobase of exon model per million mapped fragments (FPKM) values were analyzed for all genes, and the results showed that 39 genes had a high expression level in kernels with FPKM values over 500, followed by 11, 15, 14, 24, and 21, and six genes had FPKM values with 200–500, 100–200, 50–100, 20–50, 10–20, and below ten, respectively (Figure 4A). About 79% (114) of all genes expressed in kernels had higher FPKM values above 50, and few had FPKM values below 50 (Figure 4A). To better address the expression pattern, the ratios of maximal expression in all tissues and maximal expression in kernels (MaxExp/MaxExpKernel) were further analyzed. A total of 92 genes had MaxExp/MaxExpKernel values of 1, indicating that these genes expressed at the highest level in kernels but not in other tissues. The MaxExp/MaxExpKernel values with a range of one to three were for 22 genes, and three to five for six genes, over five for ten genes (Figure 4B). If the ratio of MaxExp/MaxExpKernel ≤ 3 and FPKM value of MaxExpkernel ≥ 50 were used as the filter criterion, 123 genes could be grabbed from all reported cloned genes (Figure 4C).

Furthermore, GO enrichment analysis was conducted to investigate the functions of the reported cloned genes. In the molecular function category, the most significantly enriched GO terms were “RNA binding”, “nuclease activity”, “endonuclease activity”, “zinc ion binding”, and “oxidoreductase activity, acting on paired donors” (Figure 4D). In the biological process category, reported genes were strongly enriched in the terms “RNA processing”, “seed development”, “embryo development”, “RNA splicing”, “protein complex biogenesis”, and “hormone metabolic process” (Figure 4D).

### 2.3. Characterization of QTL Clusters for Kernel Size-Related Traits in Maize

**Figure 5:**
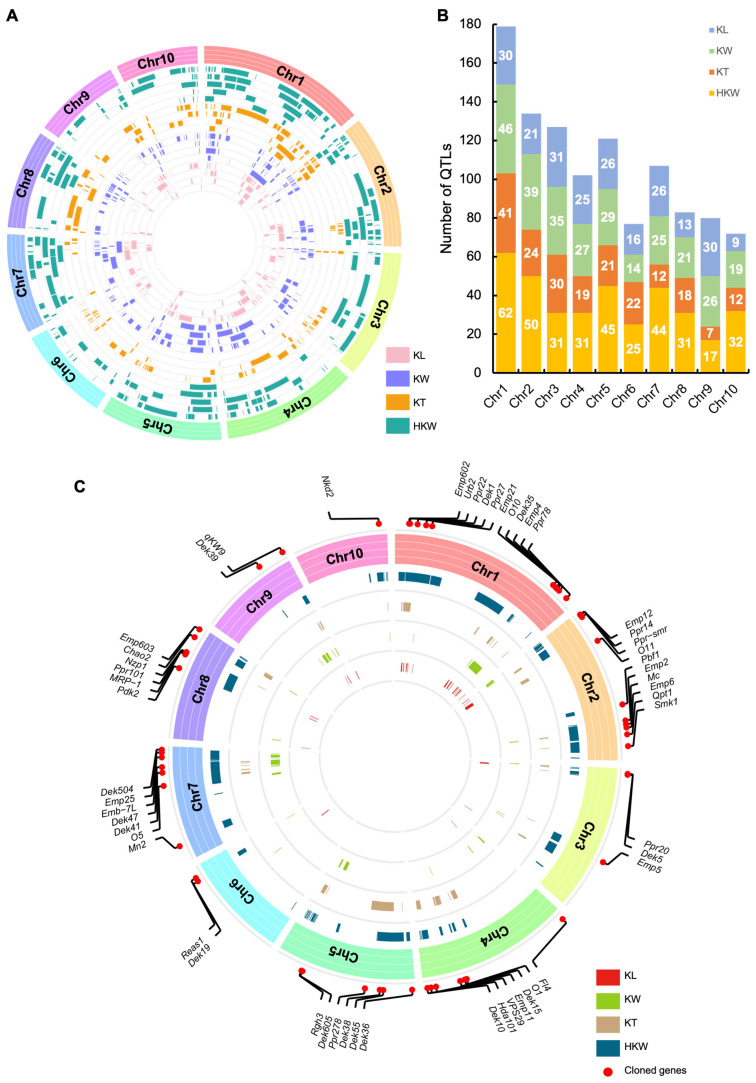
Graphic illustration of identified QTLs and QTL clusters of four maize kernel size-related traits. (**A**) Diagram of genomic distribution of original QTLs for four traits on ten maize chromosomes. (**B**) The number and distribution of original QTLs for four traits on ten maize chromosomes. (**C**) Distribution of QTL clusters and known cloned genes. KL, kernel length; KW, kernel width; KT, kernel thickness; HKW, hundred-kernel weight; Chr, chromosome.

Forty-five QTL studies on the regulation of kernel size published from 2006 to 2022 were collected from the published literature (Table S1). A total of 1456 independent QTLs for four kernel size-related traits (KL, KW, KT and HKW) were collected (Table S1). QTL projection was performed using the physical positions of flanking markers of each QTL. A total of 374 QTLs could not be projected due to the incomplete flanking marker information. Finally, 1082 QTLs, including 227 QTLs related to KL, 281 to KW, 206 to KT and 368 to HKW (Figure 5A,B), were successfully projected and used for further analysis. These QTLs were distributed randomly on the ten maize chromosomes. The total number of QTLs per chromosome ranged from 72 to 179 on chromosomes 10 and 1, respectively (Figure 5A,B). More QTLs were gathered on chromosomes 1 (179), 2 (134), and 3 (127) and fewer were on chromosomes 10 (72), 6 (77), and 9 (80) (Figure 5A,B).

Next, we conducted an assay for identification of QTL clusters for kernel size-related traits in maize. A densely populated QTL region containing at least three QTLs was defined as a QTL cluster in this study. A total of 187 QTL clusters with multiple QTLs co-localizing were identified for four kernel size-related traits (Figure 5C and Table S2). Among these QTL clusters, 38 were associated with KL, 51 with KW, 33 with KT, and 65 with HKW. QTL clusters related to each trait, except KT, were distributed on all ten maize chromosomes. Similar to QTL distribution, more QTL clusters localized on chromosomes 1 (35), 2 (27), and 3 (21) and fewer on chromosomes 10 (eight) and 6 (six) (Figure 5C and Table S2). Forty clusters harbored 67 genes known to kernel size-related traits, while the rest contained no known genes. Over half of the QTL clusters (97 out of 187) harbored ten or more QTLs, 20 QTL clusters contained 20 or more QTLs, and three had 40 or more QTLs. The highest enrichment of QTLs was identified in HKW-qCL2-13 spanning a physical length of 48.5 Mb (20,505,000–69,017,291) on chromosome 1. This QTL cluster harbored 59 QTLs and five known genes (*Emp602*, *Urb2*, *Ppr22*, *Dek1*, and *Ppr27*) associated with HKW. Another two enriched regions HKW-qCL7-3 (49) and KW-qCL1-2 (40), were identified for HKW and KW, with three (*O5*, *Dek41*, and *Dek47*) and (*Emp602*, *Urb2*, and *Ppr22*) known genes for each region, respectively (Figure 5C and Table S2). Thus, QTL clusters are highly informative and may harbor high-confidence genes for controlling kernel size-related traits.

### 2.4. Characterization of QTN Clusters for Kernel Size-Related Traits in Maize

**Figure 6:**
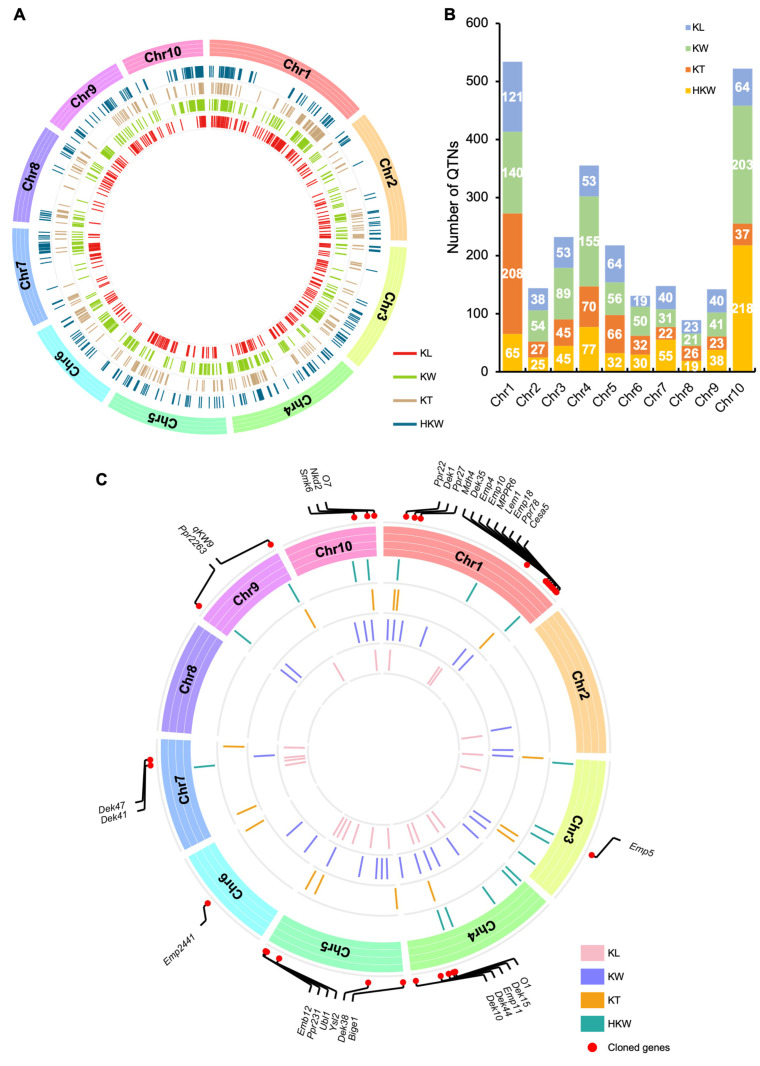
Graphic illustration of identified QTN and QTN clusters of four maize kernel size-related traits. (**A**) Diagram of genomic distribution of original QTN for four traits on ten maize chromosomes. (**B**) The number and distribution of original QTNs for four traits on ten maize chromosomes. (**C**) Distribution of QTN clusters and known cloned genes. KL, kernel length; KW, kernel width; KT, kernel thickness; HKW, hundred-kernel weight; Chr, chromosome.

Recently, GWAS has been a powerful and routine approach for identifying causal genetic variants of diverse traits in maize, including agronomic, quality, biochemical, physiological traits, and stress tolerance traits [157,158,159,160,161,162]. Through the collection of QTN data from previous studies, 2515 QTNs associated with four kernel size-related traits were extracted and successfully projected on a reference genome, among which, 515 QTNs were detected for KL, 840 for KW, 556 for KT and 604 for HKW (Figure 6A,B). These QTNs were located on all ten maize chromosomes, with more QTNs on chromosomes 1 (534) and 10 (522), and fewer on chromosome 8 (89). The common feature was that these QTNs for each trait were distributed on all ten maize chromosomes; however, the distribution density was inconsistent with each other. The highest density QTNs were detected on chromosome 1 for KL (121) and KT (208) and chromosome 10 for KW (203) and HKW (218), respectively (Figure 6A,B).

Next, the collected QTNs were submitted to identify QTN clusters. A QTN cluster in this study was admitted when five or more QTNs were co-located in this chromosomal region. The results showed that a total of 84 QTN clusters were obtained for all traits, 34 of which contained ten or more QTNs, and even three (HKW-gCL10-2, KT-gCL1-3, KW-gCL10-3) of which had more than 100 QTNs (Table S3). Among these QTN clusters, 22 for KL were distributed on chromosomes 1, 2, 3, 4, 5, 7, 9, and 10, 30 for KW were on chromosomes 1, 2, 3,4, 5, 6, 7, 9, and 10, 15 for KT were on chromosomes 1, 3, 4, 5, 6, 7, 9, and 10, 17 for HKW were on chromosomes 1,3, 4, 7, 9, 10, and none of the QTN clusters was detected on chromosome 8 (Figure 6C and Table S3). Moreover, a total of 55 cloned genes co-localized with 33 clusters, and the most enriched one was KT-gCL1-3 on chromosome 1. KT-gCL1-3 spanned a region of 29.2 Mb, containing eight cloned genes including *Dek35*, *Emp4*, *Emp10*, *MPPR6*, *Lem1*, *Emp18*, *Ppr78*, and *Cesa5* (Figure 6C and Table S3). Undoubtedly, more genes controlling yield-related traits will be discovered in these QTN clusters.

### 2.5. Integrating QTL and QTN Clusters Related to Kernel Size-Related Traits in Maize

To more accurately grasp the casual genes regulating kernel size-related traits, we further integrated the QTL/QTN clusters to identify the consensus hotspot region with at least three QTL/QTN clusters. As a result, 31 hotspot regions were identified. Chromosome 1 contained the highest number of hotspots (seven), followed by chromosomes 5 (six), 4 (five), 7 (four), 2 (three), 3 (two), 10 (two), 8 (one), and 9 (one), and chromosome 6 had no hotspots (Table 2). The number of clusters per hotspot ranged from three to 14, and 12 hotspots had five or more individual QTL/QTN clusters, suggesting that they may harbor enriched genes contributing to maize yield (Table 2). One third of hotspots (10) contained genes that have been identified to control yield-related traits, and two thirds (21) of hotspots had no cloned genes and thus, need to be further confirmed (Table 2).

The most attractive hotspot HS02 on chromosome 1 was 32 Mb in length, containing 14 QTL/QTN clusters covering four traits. Four known genes (*Emp602*, *Urb2*, *ppr22*, and *Dek1*) were identified to be co-located in HS02. The HS07 on chromosome 1 had six known genes (*Dek35*, *Emp4*, *Emp10*, *MPPR6*, *Lem1*, and *Emp18*), the most known genes gathering on a particular overlapped region. HS11 on chromosome 3 had eight clusters, but no known genes were identified in this hotspot. Similarly, no genes have been detected in HS06, HS09, HS12, HS20, and HS22 hotspots harboring high numbers of clusters (Table 2). Thus, more attention could be paid to those hotspots without known genes or with limited genes.

### 2.6. Identification of Candidate Genes Controlling Kernel Development in Maize

Owing to the critical roles in maize kernel developmental process, PPR genes were first searched for 31 identified hotspots. A total of 85 new PPR genes were detected, whose roles in kernel development remain to be investigated in further studies (Table S6).

We also tried to screen other regulatory factors for these hotspot regions. As many hotspots were identified in this study, we chose six attractive hotspot regions (OL02, OL06, OL09, OL11, OL26, and OL31), harboring high numbers of QTL/QTN clusters or without reported genes, as examples for further candidate gene analysis. Based on the physical positions, a total of 2634 genes were collected for the six hotspot regions. After filtering by the union condition of MaxExp/MaxExpKernel ≤ 3 and MaxExpKernel ≥ 50, 1314 genes were extracted for further GO enrichment analysis (Table S4). We focused on four GO terms, including “RNA processing”, “hormone metabolic process”, “starch metabolic process”, and “mitochondrial RNA metabolic process”, which were involved in kernel development pathways [6,33]. Finally, a total of 148 genes with no PPR genes were hypothesized as candidate genes controlling kernel size-related traits in maize (Table S5). The roles of these genes also required further investigation by experiments.

## 3. Discussion

Kernel size-related traits are genetically complex quantitative traits. QTL mapping and GWAS analysis methods have provided a huge amount of information for the traits. However, progress in fine mapping of causal genes and utilization of them in maize breeding programs is limited because of little systematical intergradation and validation of QTLs and QTNs. Hotspot analysis is an effective method for optimization and validation of published QTLs and QTNs, identifying true QTLs and QTNs via accurate consensus regions. Thus, a comprehensive study based on published information is required and was addressed in this study.

The complexity of maize kernel size-related traits refers to not only multiple loci controlled but also intricate regulatory networks involved. It has been well documented that cloned genes controlling maize kernel size largely encode for PPR proteins, belonging to a large family of nucleic acid binding proteins, mainly, RNA-binding proteins. PPR proteins play multiple roles in many biological processes in organelles, including transcription, RNA stabilization, RNA cleavage, translation, RNA splicing, and RNA editing, thereby affecting the expression of organelle genes [163]. Mutations in maize PPR proteins are commonly associated with severe defects in kernel development as summarized in Table 1. Starch is the major component of maize kernels; thus, genes participating in the starch metabolic process may affect kernel filling process, such as *Ae1* [126], *Bt2* [127], *Se1* [128], *Sh2* [129], *Su1* [130], *SWEET4c* [106], *Dof3* [70], *Incw1* [143], *Mn1* [11], and *Mn6* [13] (Table 1). Plant hormone-related genes have also been found to control the kernel development in maize, including an auxin homeostasis regulatory gene *Ehd1* [37] and a brassinosteroid biosynthesis gene *Drg10* [133] (Table 1). Moreover, transcription factors also play critical roles in kernel development in maize. *OPAQUE11* (*O11*) functions as a central hub of the endosperm regulatory network connecting storage reserve accumulation and metabolism, stress responses, and endosperm development [6].

Several previous studies have integrated QTL or QTN data to find more informative loci. For example, a QTL consistency and meta-analysis identified that 16 meta-QTLs from 138 QTLs for eight grain yield components in three generations were derived from the same two parents [164]. Wang et al. tried to combine meta-QTL and GWAS raw signals to dissect candidate genes for maize yield [34]. In this study, we first did three integration analysis to characterize QTL clusters, QTN clusters, and consensus hotspot regions of both. The final identified genomic regions were more informative and highly confident for predicting candidate genes.

Candidate gene analysis can be carried out based on GO enrichment annotations [13], expression pattern [34], and homologous genes in species and inter species [165,166]. Here, we established a new approach, a combination of expression pattern and GO annotations, to quickly extract candidate genes for kernel development from large gene pools. This will be helpful for prediction of candidate genes from not only hotspot regions but also newly detected genomic regions. Even though, we still could not exclude the possibility that some genes might indirectly function in controlling kernel size with no expression in kernel tissues. Here, it should be noted that we only performed candidate gene analysis for six representing hotspots. Further analysis is required for the other hotspots, QTL and QTN clusters not within any hotspot region.

**Figure 7:**
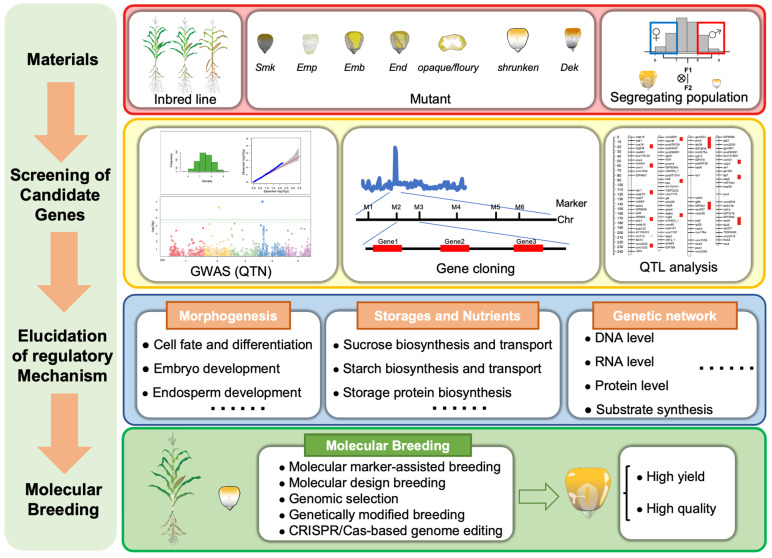
A strategy of how to produce a new generation of high yield and quality maize through molecular breeding. *Smk*: *small kernel* mutants; *Emp*: *empty pericarp* mutants; *Emb*: *embryo specific* mutants; *End*: *endosperm specific* mutants; *opaque/floury*: *opaque/floury* mutants; *shrunken*: *shrunken* mutants; *Dek*: *defective kernel* mutants.

The final goal of studies on kernel size-related traits in maize is to identify elite genes for improving maize yield. Here, we proposed a strategy model for the generation of high yield and quality maize, following a path as follows (Figure 7): Step 1, Collection of various plant materials, such as inbred lines, mutants and segregating populations in diverse genetic backgrounds; Step 2: Screening and identification of candidate genes via QTL mapping, GWAS analysis, and other gene cloning methods; Step 3: Elucidation of regulatory mechanisms controlling morphogenesis, storages, and nutrients to build genetic networks at different regulatory levels; and Step 4: Molecular breeding of high yield and quality maize by molecular marker-assisted breeding, molecular design breeding, genomic selection, genetically modified breeding, and CRISPR/Cas-based genome editing technologies [167,168,169,170,171]. This strategy can be also adopted to develop maize cultivars with other qualities of interest, for instance, biotic and abiotic tolerance and resistance.

## 4. Materials and Methods

### 4.1. Bibliometric Analysis

Bibliometric analysis was conducted based on a series of searching queries in Web of Science, after reviewing the keywords and abstracts of related publications. A total of 952 research articles and reviews were retrieved, with records of authors, affiliated institutions, publication journals, years, titles, and abstracts, spanning literature published between 2000 and 2022 (up to 25 November 2022). Finally, the valid papers were analyzed for specific bibliometric indicators including publication volume, keywords, and high-frequency words, then visualized with CiteSpace (Drexel University, Philadelphia, PA, USA) and Scimago Graphica (SCImago Lab, Granada, Spain).

### 4.2. Gene, QTL and QTN Data Collection

An exhaustive bibliographic review was performed on maize cloned genes, QTLs and QTNs related to four kernel size-related traits (KL, KW, KT, and HKW). A total of 132 cloned genes were summarized in Table 1. A total of 45 QTL studies published were extracted for QTL data collection, and 14 GWAS publications were for QTNs data collection. The basic information of each literature was collected, including “trait type”, “population type”, “population size”, “number of environments”, “mapping method”, “chromosomal position”, “markers”, “proportion of variance explained (R^2^)”, “confidence interval”, and “limit of detection (LOD) value”, parts of which were listed in Table S1.

### 4.3. Projection of QTL, QTNs, and Genes on Reference Genome

QTL projection was carried out using flanking markers of the collected QTLs. QTLs were projected on B73 reference genome sequence V4 (B73_V4, http://maizeGDB.org, accessed on 30 November 2022). All collected QTNs and target genes were also projected on B73_V4 based on their physical positions.

### 4.4. Identification of QTL and QTN Clusters

After projection, QTL cluster analysis was performed by a powerful toolset Bedtools (https://bedtools.readthedocs.io/en/latest/index.html, accessed on 30 November 2022). A genomic region was defined as a QTL cluster if at least three QTLs were co-localized. QTN cluster analysis was done manually by searching in a sliding window of 5 Mb on each chromosome, and a QTN cluster region was approved if this region harbored at least five QTNs. QTL and QTN clusters for each trait were searched on all ten maize chromosomes and designated as “Trait-qCL-chromosome-number” in Table S2 and “Trait-gCL-chromosome-number” in Table S3, respectively.

### 4.5. Integration of QTL and QTN Hotspots

Based on physical positions, QTL and QTN clusters were integrated with the toolkit Bedtools to discover the consensus regions. A QTL/QTN hotspot was defined if at least three QTL or QTN clusters were co-localized. QTL/QTN hotspot was designated as “HS-number”. A total of 31 QTL/QTN hotspots were summarized and listed in Table 2. Gene models in hotspot regions were extracted from MaizeGDB based on the physical positions and submitted to further GO enrichment and gene expression analysis.

### 4.6. GO Enrichment Analysis

GO enrichment analysis was performed using a web-based server agriGO2.0 (http://systemsbiology.cau.edu.cn/agriGOv2/index.php#, accessed on 30 November 2022) [172]. The investigated genes were assigned to GO categories for Molecular Function and Biological Process.

### 4.7. In Silico Gene Expression Analysis

In Silico Gene expression analysis was performed using an RNA-seq resource published previously [156]. Expression patterns of cloned and candidate genes associated with kernel size-related traits were investigated in this study.

## 5. Conclusions and Prospects

Nowadays, maize yield is challenged by population growth, various biotic and abiotic stresses, and climate change. Therefore, it is important to enhance our understanding of the genomic architecture of kernel size-related traits controlling maize yield. The cluster analysis revealed that a total of 187 QTL clusters were identified for KL, KW, KT, and HKW traits, while 84 QTN clusters were detected for the kernel size-related traits. Moreover, 31 consensus hotspot regions contained multiple QTL and QTN clusters for controlling kernel size-related traits. Candidate gene analysis revealed that 85 PPR genes were detected in QTL/QTN hotspots and 148 other candidates were predicted for six attractive hotspots. The characterization of cloned genes in expression patterns could significantly strengthen the exploitation of candidate genes in undeveloped genomic regions. The identified hotspot regions and candidate genes provided useful resources for molecular breeding to improve maize yield.

## Figures and Tables

**Table 1:** Summary of cloned genes involved in maize kernel size.

Phenotype ^a^	Gene Name	Gene ID (B73_V4)	Chr	Gene Annotation	Reference
*Dek*	*Arm4*	Zm00001d053964	4	ARM repeat protein, unknown pathway	[35]
	*Dsc1*	Zm00001d049871	4	ARF-GTPases, vesicular material transport	[36]
	*Ehd1*	Zm00001d053858	4	EHD proteins, regulation of auxin homeostasis	[37]
	*Qpt1*	Zm00001d006311	2	Quinolinate phosphoribosyltransferase, vitamin B biosynthesis	[38]
	*Rgh3*	Zm00001d016836	5	U12 splicing factor, U12-type intron splicing	[39]
	*Skus1*	Zm00001d043090	3	Multi-copper oxidase, regulation of redox homeostasis	[40]
	*Dek53*	Zm00001d041326	3	PPR protein, RNA editing	[41]
	*Nzp1*	Zm00001d010994	8	Mitochondrial 50S ribosomal protein L10, protein body formation, mitochondrial complex assembly	[42]
	*Reas1*	Zm00001d038475	6	Ribosome export associated1, ribosome biosynthesis	[43]
	*Dek47*	Zm00001d021372	7	RCC1 domain-containing protein RUG3, RNA splicing	[44]
	*Dek48*	Zm00001d002539	2	PPR protein, RNA editing	[45]
	*Dek504*	Zm00001d022394	7	PPR protein, RNA editing	[46]
	*Dek55*	Zm00001d014471	5	PPR protein, RNA splicing and editing	[47]
	*Dek1*	Zm00001d028818	1	Membrane protein, plant signal transduction	[48]
	*Dek10*	Zm00001d053802	4	PPR protein, RNA editing	[49]
	*Dek15*	Zm00001d052197	4	Cohesin-loading complex subunit SCC4, ensuring proper chromosome segregation	[50]
	*Dek19*	Zm00001d038257	6	PPR protein, unknown pathway	[51]
	*Dek2*	Zm00001d034882	1	PPR protein, RNA splicing	[52]
	*Dek33*	Zm00001d016475	5	Pyrimidine reductase, riboflavin biosynthesis	[53]
	*Dek35*	Zm00001d033749	1	PPR protein, RNA splicing	[54]
	*Dek36*	Zm00001d013136	5	PPR protein, RNA editing	[55]
	*Dek37*	Zm00001d003543	2	PPR protein, RNA splicing	[56]
	*Dek38*	Zm00001d014595	5	Tel2-interacting protein 2, promoting early seed development through the action of PIKKs	[57]
	*Dek39*	Zm00001d047013	9	PPR protein, RNA editing	[58]
	*Dek40*	Zm00001d011478	8	PBAC4 Protein, ubiquitin-20S Proteasome Biogenesis	[59]
	*Dek41*	Zm00001d021053	7	PPR protein, RNA splicing	[60]
	*Rbm48*	Zm00001d054077	4	RNA-binding protein, pre-mRNA spliceosome formation	[61]
	*Dek44*	Zm00001d052865	4	Mitochondrial ribosomal protein L9, well-functioning in oxidative phosphorylation	[62]
	*Dek45*	Zm00001d023331	10	PPR protein, RNA editing	[63]
	*Dek46*	Zm00001d043107	3	PPR protein, RNA editing	[64]
	*Dek5*	Zm00001d039612	3	*E. coli* TamB homologous, chloroplast envelope biogenesis;	[65]
	*Dek605*	Zm00001d016798	5	PPR protein, RNA editing	[66]
	*MPPR6*	Zm00001d034111	1	PPR protein, facilitating translation initiation	[67]
	*Nkd1*	Zm00001d002654	2	IDD transcription factors, central regulators of gene expression in endosperm development	[68]
	*Nkd2*	Zm00001d026113	10	IDD transcription factors, central regulators of gene expression in endosperm development	[68]
	*Shai1*	Zm00001d002661	2	RWP-RK transcription factor, embryo polarity establishment, polar transport of IAA	[69]
	*Dof3*	Zm00001d035651	6	Dof-type transcription factor, starch accumulation and aleurone layer development	[70]
*Emb*	*Bige1*	Zm00001d012883	5	MATE-type transporter, CYP78A pathway (transport of growth factors)	[71]
	*Emb12*	Zm00001d018366	5	Translation initiation factor 3, plastid protein synthesis	[72]
	*Emb14*	Zm00001d054079	4	Plastid-targeted cGTPase, ribosome formation in plastid	[73]
	*Why1*	Zm00001d036148	6	DNA/RNA binding protein, genome stabilization and ribosome formation in plastids	[74]
	*Emb-7L*	Zm00001d021871	7	Plastid PPR protein, RNA splicing	[75]
	*Lem1*	Zm00001d034192	1	Plastid 30S ribosomal protein S9, maintenance of plastid stability and ribosome formation	[76]
	*PPR8522*	Zm00001d034962	1	Plastid PPR protein, chloroplast transcription	[77]
	*PRPL35-1*	Zm00001d046555	9	Plastid ribosomal L35 subunit, translation	[78]
*Emp*	*Emp2441*	Zm00001d036689	6	Nuclear-encoded maturase 3 protein, RNA splicing	[79]
	*Ppr14*	Zm00001d002157	2	PPR protein, RNA splicing	[80]
	*Ppr22*	Zm00001d028422	1	PPR protein, RNA editing	[81]
	*Ppr166*	Zm00001d040222	3	PPR protein, RNA editing	[82]
	*Mcsf1*	Zm00001d024429	10	CRM domain-containing protein, interaction with PPR protein	[83]
	*Ppr-smr*	Zm00001d002345	2	PPR protein, RNA splicing	[83]
	*Emp25*	Zm00001d022184	7	PPR protein, RNA splicing	[84]
	*Emp603*	Zm00001d012528	8	PPR protein, RNA splicing	[85]
	*Emp80*	Zm00001d009677	8	PPR protein, RNA editing	[86]
	*Emp11*	Zm00001d052450	4	PPR protein, RNA splicing	[87]
	*Emp12*	Zm00001d002098	2	PPR protein, RNA splicing	[88]
	*Emp16*	Zm00001d011559	8	PPR protein, RNA splicing	[89]
	*Emp18*	Zm00001d034253	1	PPR protein, RNA editing	[90]
	*Emp10*	Zm00001d033992	1	PPR protein, RNA splicing	[91]
	*Emp2*	Zm00001d005675	2	Heat shock binding protein 1, heat shock response	[92]
	*Emp21*	Zm00001d033495	1	PPR protein, RNA editing	[93]
	*Emp32*	Zm00001d040363	3	PPR protein, RNA splicing	[94]
	*Emp4*	Zm00001d033869	1	PPR protein, correct expression of mitochondrial transcripts	[95]
	*Emp6*	Zm00001d005959	2	PORR protein, mitochondrial intron splicing	[96]
	*Emp602*	Zm00001d028046	1	PPR protein, RNA splicing	[97]
	*Emp7*	Zm00001d008298	8	PPR protein, RNA editing	[98]
	*Emp8*	Zm00001d049796	4	PPR protein, RNA splicing	[99]
	*Emp9*	Zm00001d022480	7	PPR protein, RNA editing	[100]
	*Ppr101*	Zm00001d010942	8	PPR protein, RNA splicing	[101]
	*Ppr27*	Zm00001d029061	1	PPR protein, RNA editing	[82]
	*Ppr18*	Zm00001d007927	2	PPR protein, RNA splicing	[102]
	*Ppr20*	Zm00001d039548	3	PPR protein, RNA splicing	[103]
	*Emp5*	Zm00001d042039	3	PPR protein, RNA editing	[104]
	*Sal1*	Zm00001d046599	9	Lass E vacuolar sorting protein, aleurone layer differentiation	[105]
	*SWEET4c*	Zm00001d015912	5	Bidirectional sugar transporter SWEET4-like, hexose transport	[106]
*End*	*Cesa5*	Zm00001d034553	1	Cellulose synthase 5, flange cell wall ingrowths formation	[107]
	*Mn6*	Zm00001d037926	6	ER SPases I, signal cleavage	[13]
	*Cr4*	Zm00001d023425	10	Receptor-like kinase, cell differentiation	[108]
	*De18*	Zm00001d023718	10	Endosperm-specific YUCCA1 protein, IAA biosynthesis	[109]
	*Mdh4*	Zm00001d032695	1	Cytosolic malate dehydrogenase 4, interconversion between malic acid and oxaloacetic acid (OAA)	[110]
	*O11*	Zm00001d003677	2	bHLH transcription factor, important regulators of endosperm development and metabolism	[111]
*opaque/floury*	*Ocd1*	Zm00001d008739	8	Oxalyl-CoA decarboxylase, oxalate degradation	[112]
	*Fl1*	Zm00001d003398	2	Endoplasmic reticulum protein, protein body assembly	[113]
	*Fl2*	Zm00001d049243	4	22-kD a-zein protein, zein biosynthesis	[114]
	*Fl3*	Zm00001d009292	8	PLATZ protein, tRNA and 5S rRNA transcription	[115]
	*Fl4*	Zm00001d048851	4	19-kD a-zein z1A-6, protein body assembly	[116]
	*Mc*	Zm00001d005793	2	16-kD-*γ*-zein, zein biosynthesis	[117]
	*O1*	Zm00001d052110	4	Myosin XI motor protein, morphology and movement of the endoplasmic reticulum, protein body formation	[118]
	*O10*	Zm00001d033654	1	Novel cereal-specific protein, regulation of protein distribution	[119]
	*O2*	Zm00001d018971	7	bZIP transcription factor, multiple biological process regulators in the endosperm	[15]
	*O5*	Zm00001d020537	7	Monogalactosyldiacylglycerol synthase, MGDG biosynthesis	[120]
	*O6/Pro1*	Zm00001d010056	8	P5CS, proline biosynthesis	[121]
	*O7*	Zm00001d026649	10	Acyl-activating enzyme, zein biosynthesis	[122]
	*Pbf1*	Zm00001d005100	2	Prolamin-box binding factor, regulation of zein expression	[123]
	*Pdk1*	Zm00001d038163	6	Pyruvate phosphate dikinase, energy production and metabolism	[124]
	*Pdk2*	Zm00001d010321	8	Pyruvate phosphate dikinase, energy production and metabolism	[124]
	*Smu2*	Zm00001d023239	10	RNA-splicing factor, rRNA processing and protein synthesis	[125]
*shrunken*	*Ae1*	Zm00001d016684	5	Starch-branching enzyme IIb, starch biosynthesis	[126]
	*Bt2*	Zm00001d050032	4	ADP-glucose pyrophosphorylase, starch biosynthesis	[127]
	*Se1*	Zm00001d007657	2	FAF domain protein, starch biosynthesis	[128]
	*Sh1*	Zm00001d045042	9	Sucrose synthase, starch biosynthesis	[16]
	*Sh2*	Zm00001d044129	3	AGPase subunit, starch biosynthesis	[129]
	*Su1*	Zm00001d049753	4	Isoamylase, starch biosynthesis	[130]
	*NAC128*	Zm00001d040189	3	NAC transcription factor, starch and zein biosynthesis	[131]
	*NAC130*	Zm00001d008403	8	NAC transcription factor, starch and zein biosynthesis	[131]
*Smk*	*Chao2*	Zm00001d011819	8	Chlorophyll a oxygenase 1, chlorophyll B synthesis	[132]
	*Drg10*	Zm00001d003349	2	Cytochrome P450 protein, brassinosteroid biosynthesis	[133]
	*Expb14*	Zm00001d045792	9	Expansin protein, miR164 pathway, participating in kernel expansion	[134]
	*Expb15*	Zm00001d045861	9	Expansin protein, miR164 pathway, participating in kernel expansion	[134]
	*qKW9*	Zm00001d048451	9	Plastid PPR protein, RNA editing	[135]
	*Ppr78*	Zm00001d034428	1	PPR protein, nad5 mature and mRNA stabilization	[136]
	*Ppr278*	Zm00001d015156	5	PPR protein, RNA splicing and editing	[137]
	*Smk1*	Zm00001d007100	2	PPR protein, RNA editing	[138]
	*Smk10*	Zm00001d001803	2	Choline transporter-like protein, choline transport pathway	[139]
	*Smk501*	Zm00001d008256	8	RUBylation activating enzyme E1 subunit ECR1, ubiquitin-related RUB pathway	[140]
	*Vks1*	Zm00001d018624	7	Kinesin-14 motor protein, regulation of mitosis and cytokinesis	[141]
	*Mn2*	Zm00001d019294	7	Nitrate transporter, bidirectional transport of nitrate	[142]
	*Incw1*	Zm00001d016708	5	Cell wall invertases 1, sucrose cleavage and transport	[143]
	*Hda101*	Zm00001d053595	4	Histone deacetylase, maintenance of histone acetylation	[144]
	*Mn1*	Zm00001d003776	2	Cell wall isozymes 2, sucrose cleavage and transport	[11]
	*MRP-1*	Zm00001d010889	8	Transfer cell-specific transcriptional activator, regulator of the differentiation of transfer cells	[145]
	*Ppr231*	Zm00001d018219	5	PPR protein, RNA splicing	[101]
	*Ppr2263*	Zm00001d045089	9	PPR protein, RNA editing	[146]
	*VPS29*	Zm00001d053371	4	Retromer complex subunit, regulation of IAA homeostasis	[147]
	*Smk2*	Zm00001d053981	4	Glutaminase, vitamin B6 Biosynthesis	[148]
	*Smk3*	Zm00001d041537	3	Mitochondrial transcription termination factor, intron splicing and complex assembly	[149]
	*Smk4*	Zm00001d049196	4	PPR protein, RNA editing	[150]
	*Smk6*	Zm00001d025446	10	PPR protein, RNA editing	[151]
	*Smk7*	Zm00001d035960	6	RNA polymerase III subunit, transcriptional regulation of tRNA and 5s rRNA	[152]
	*Ubl1*	Zm00001d017432	5	Putative RNA exonuclease, pre-mRNA splicing	[153]
	*Urb2*	Zm00001d028096	1	Urb2 domain-containing protein, pre-ribosomal RNA processing	[154]
	*Ysl2*	Zm00001d017427	5	Iron-nicotianamine transporter, Fe stabilization and storage	[155]

^a^*Dek: defective kernel* mutants; *Emp: empty pericarp* mutants; *Emb: embryo specific* mutants; *End: endosperm specific* mutants; *Smk: small kernel* mutants; *opaque/floury: opaque/floury* mutants; *shrunken: shrunken* mutants.

**Table 2:** Summary of QTL/QTN hotspots associated with kernel size-related traits in maize.

ID	Chr	Physical Position (B73_V4, nt)	Overlapped Cluster		Cloned Gene	
			Number	Detail	Number	Detail
HS01	1	12,622,245–17,191,112	3	KW-gCL1-1, KW-qCL1-1, HKW-qCL1-1	0	
HS02	1	20,505,000–52,520,534	14	KW-qCL1-2, HKW-qCL1-2, KT-qCL1-1, KL-gCL1-1, KT-gCL1-1, KT-qCL1-2, KT-qCL1-3, HKW-gCL1-1, KW-gCL1-2, KT-qCL1-4, KT-gCL1-2, KT-qCL1-5, KT-qCL1-6, KT-qCL1-7	4	*Emp602*, *Urb2*, *Ppr22*, *Dek1*
HS03	1	53,029,436–59,415,031	3	KW-gCL1-3, KL-qCL1-1, HKW-qCL1-2	1	*Ppr27*
HS04	1	211,711,329–221,020,719	3	KL-gCL1-2, KT-qCL1-9, HKW-qCL1-4	0	
HS05	1	238,107,995–242,396,593	4	KL-gCL1-3, KL-qCL1-2, KT-qCL1-11, KW-qCL1-5	0	
HS06	1	244,038,128–252,279,335	6	KW-qCL1-5, HKW-qCL1-5, KL-qCL1-3, KW-gCL1-5, KW-qCL1-6, HKW-qCL1-6	0	
HS07	1	270,393,381–288,290,428	6	KL-qCL1-3, KT-gCL1-3, KW-gCL1-6, KT-qCL1-15, KT-qCL1-16, HKW-gCL1-3	6	*Dek35*, *Emp4*, *Emp10*, *MPPR6*, *Lem1*, *Emp18*
HS08	2	1,645,703–3,317,858	3	KL-qCL2-1, HKW-qCL2-1, KT-qCL2-1	0	
HS09	2	19,436,743–33,434,209	5	KW-qCL2-1, HKW-qCL2-7, KT-qCL2-3, KW-qCL2-2, KW-qCL2-3	0	
HS10	2	193,515,365–196,000,000	3	KW-qCL2-5, KL-qCL2-4, HKW-qCL2-11	1	*Emp6*
HS11	3	1,325,039–3,615,750	8	KW-gCL3-1, KT-gCL3-1, KL-gCL3-1, KW-qCL3-1, KL-qCL3-1, KL-qCL3-2, KW-qCL3-2, HKW-qCL3-1	0	
HS12	3	4,388,286–6,099,395	5	KL-gCL3-1, KW-qCL3-3, HKW-qCL3-2, KT-qCL3-1, KW-qCL3-4	0	
HS13	4	3,561,183–6,010,197	3	KL-qCL4-1, HKW-gCL4-1, KW-gCL4-1	0	
HS14	4	158,189,789–163,298,733	4	KW-gCL4-4, HKW-gCL4-4, KW-qCL4-2, KL-gCL4-3	0	
HS15	4	175,915,845–193,659,742	8	KT-gCL4-1, HKW-gCL4-5, HKW-qCL4-3, KL-gCL4-4, KW-qCL4-3, KL-qCL4-3, KW-qCL4-4, KW-gCL4-5	3	*O1*, *Dek15*, *Emp11*
HS16	4	196,013,048–201,338,547	3	HKW-qCL4-4, KW-qCL4-6, KW-gCL4-5	0	
HS17	4	237,957,206–240,000,000	3	KW-qCL4-7, KW-gCL4-6, HKW-qCL4-6	0	
HS18	5	14,156,572–16,289,485	3	KW-qCL5-1, HKW-qCL5-2, KL-gCL5-1	0	
HS19	5	34,324,375–65,639,673	4	KW-qCL5-2, KW-gCL5-2, KW-gCL5-3, HKW-qCL5-2	2	*Dek36*, *Dek55*
HS20	5	188,839,192–195,089,478	5	HKW-qCL5-6, HKW-qCL5-5, KW-gCL5-5, KT-gCL5-2, KL-gCL5-4	0	
HS21	5	196,011,766–199,615,219	3	KW-qCL5-3, HKW-qCL5-7, KL-qCL5-2	0	
HS22	5	202,544,328–209,785,812	5	HKW-qCL5-11, HKW-qCL5-10, KL-gCL5-5, HKW-qCL5-9, KW-qCL5-4	0	
HS23	5	211,000,000–212,583,743	3	HKW-qCL5-12, KT-gCL5-3, KL-gCL5-5	0	
HS24	7	112,866,130–118,353,636	3	HKW-qCL7-3, KL-qCL7-1, KL-gCL7-1	0	
HS25	7	125,132,474–129,166,008	4	HKW-qCL7-3, KL-gCL7-2, KW-qCL7-1, KW-qCL7-2	0	
HS26	7	132,311,932–156,631,694	11	HKW-gCL7-1, KW-qCL7-4, KL-gCL7-3, KL-qCL7-2, KL-qCL7-3, KL-qCL7-4, KW-qCL7-5, KW-gCL7-1, KW-qCL7-6, KW-qCL7-7, HKW-qCL7-3	2	*Dek41*, *Dek47*
HS27	7	172,547,374–174,900,000	4	HKW-qCL7-5, KT-gCL7-2, KL-gCL7-4, KL-qCL7-8	0	
HS28	8	167,000,000–170,036,314	3	KW-qCL8-2, KL-qCL8-3, HKW-qCL8-2	0	
HS29	9	149,400,961–157,000,000	9	HKW-qCL9-2, KL-gCL9-1, KW-qCL9-2, HKW-gCL9-2, KW-qCL9-3, KT-gCL9-1, KL-qCL9-8, KW-qCL9-4, KW-qCL9-5	1	*qKW9*
HS30	10	109,814,884–122,563,176	3	HKW-gCL10-1, KW-gCL10-2, HKW-qCL10-1	1	*Smk6*
HS31	10	130,739,025–149,279,019	10	HKW-qCL10-2, KW-gCL10-3, HKW-gCL10-2, KL-gCL10-1, KT-gCL10-1, HKW-qCL10-3, KW-qCL10-1, HKW-qCL10-4, KL-qCL10-1, HKW-qCL10-5	1	*Nkd2*

## Data Availability

Not applicable.

## Supplementary Materials

The following supporting information can be downloaded at: https://www.mdpi.com/article/10.3390/ijms24021025/s1.
